# The Hernia ‘CAMP’ model: a collaborative action to maximise productivity within the NHS

**DOI:** 10.1007/s10029-020-02121-w

**Published:** 2020-01-29

**Authors:** R. M. Koshy, M. Ali, T. Fernando, V. S. Menon

**Affiliations:** Department of General Surgery, University Hospitals of Coventry and Warwickshire NHS Trust, Coventry, CV2 2DX UK

**Keywords:** Hernia CAMP, Quality, Productivity, Hernia surgery

## Abstract

**Background:**

An ever-growing and long surgical waiting list is a challenge within the NHS. Long waiting times can result in complications of the condition with more challenging operations and additional procedures. All of which implies reduced quality of life for patients and increased strain on NHS finances. On an average there are about 160 patients on the waiting list for groin hernia surgeries, with over a half of them waiting more than 30 weeks. Three patients every year breach the 52 weeks timeline, flagging a never event, with negative implications for the trust.

**Methods:**

The Hernia CAMP model was proposed to improve productivity and enhance patient experience. It helped create a pathway with experienced non-consultant surgeons, stepping up to free up consultants to attend to the pressing cancer and complex cases. This dedicated pathway, improved the patient experience and staff team-spirit too.

**Results:**

The Hernia CAMP resulted in a 40% improvement in efficiency. With better ratio per list/session, it makes care more cost-effective. It also improved the work environment amongst staff and rapport with patients. The patient-peer support and greater involvement meant better overall experience too. This supportive environment also has the potential for theme-based learning and training.

**Conclusions:**

The Hernia 'CAMP' is a transferable and adaptable model. It impacts not just long waiting lists, but also improves productivity with definite cost benefits, teambuilding, patient experience and creates a great opportunity to train too.

## Introduction

Majority of Surgical departments across the NHS are plagued by long waiting lists and prolonged waiting times for surgery [1]. The lists are inundated with inguinal hernias and Cholecystectomies being the most common of the procedures. With cancer and complex cases prioritised, these benign cases just have to wait. The challenge and the pressure to meet the Referral-to-Treatment (RTT) targets are immense and almost impossible.

In our trust, on an average there are about 160 patients on the waiting list for an inguinal hernioplasty. The impossible RTT of 18 weeks often gets breached as the median waiting times are 26 weeks (6.5 months). About three patients breach the 52 week target on an average every year.

However, within this problem lies a solution too. Appropriately chosen cases can be delegated to senior non-consultant surgeons. This approach frees up the consultants for the prioritised cases, while stepping up responsibility for the non-consultant colleagues.

The Hernia ‘CAMP’ (Collaborative Action to Maximise Productivity) model is a team-effort that aims to tackle long waiting lists and waiting times. The aim of these two CAMPs conducted over 5 months, was to demonstrate a model for improvement and a pattern for change. In addition to improvements in efficiency and productivity, it has also improved patient involvement and experience; and improved team-spirit and morale amongst staff.

## Methods

The CAMP initiative was modelled on improving the patient pathway and efficiency by,

1. Selecting the appropriate patient for the appropriate procedure (open inguinal hernia in day surgery unit-DSU)
2. Improving the consenting process and reducing the number of visits (group patient information sessions-PIS and pre-op assessments done at the same time)
3. Forming a team, led by a consultant surgeon, non-consultant surgeons, anaesthetists and supporting staff (Nurses, ODPs, HCAs and Administrative staff)
4. Dedicated day in DSU for the CAMP
5. Feedback and follow-up.

The impact of the CAMPs was recorded by the fall in waiting times, ratio per list and patient/staff surveys. The data from the feedback were analysed to identify areas of improvement for the future CAMPs.

As this was a process improvement strategy, it needed no ethical clearances and had no conflict of interests.

The CAMP process: From the initial planning stage, the CAMP takes about 6 weeks to execution. This process is spearheaded by a surgeon, who has to have like-minded surgical and anaesthetic colleagues to build up a team for the success of the CAMP. It offers a great opportunity to collaborate with different levels of staff, from administrative to HCAs. Getting other consultant surgeons on board is crucial to the initial planning. As, one, will have to take responsibility for the process, to oversee the execution, including the operations and post-op complications if any.

The surgeon’s experience: The experience of the non-consultant surgeons involved was crucial to make the effective delivery of the CAMP a success with minimum morbidity. They were either an ‘Associate Specialist’ with more than 20 years of operative experience, senior fellow with more than 10 years of operative experience and senior registrars with about 5 years of operative experience.

The operative procedure: All the surgeons performed a standard open tension-free Lichtenstein mesh repair with a standard light-weight mesh. Most patients had uncomplicated inguinal hernias, but for two patients who had large inguino-scrotal hernias; which were all repaired the standard way as described. The one female with a femoral hernia, had a pre-peritoneal mesh repair using the modified McEvedy approach. With uncomplicated procedures, lasting an hour (mean 64 min), all but two patients were discharged home the same day. Two elderly men stayed overnight due to urinary retention issues, and were discharged the following day (still 24 h).

**Table:** 

Hernia CAMPs	Patient demographics				Types of hernioplasties performed	
	Males	Females	Age in years (Median)	ASA (Max)	Inguinal hernias	Femoral hernias
CAMP 1	12	0	56	2	9 (Right) 3 (Left)	0
CAMP 2	10	1	65	2	7 (Right) 3 (Left)	1 (Right)

The patient demographics: All the 23 patients chosen as per day surgery guidelines, (by the surgeons) were then screened (by the anaesthetists) with a maximum ASA grade 2, being the cutoff.

The post-op follow-up: All patients were followed-up with a telephonic questionnaire at 4 weeks post-op to record their experience through the new CAMP model, obtain feedback to make improvements and record post-op morbidity.

This quality improvement report was built on the SQUIRE framework, for reporting healthcare improvements. The reports of system level work and improvements made due to the intervention were all recorded as per these guidelines.
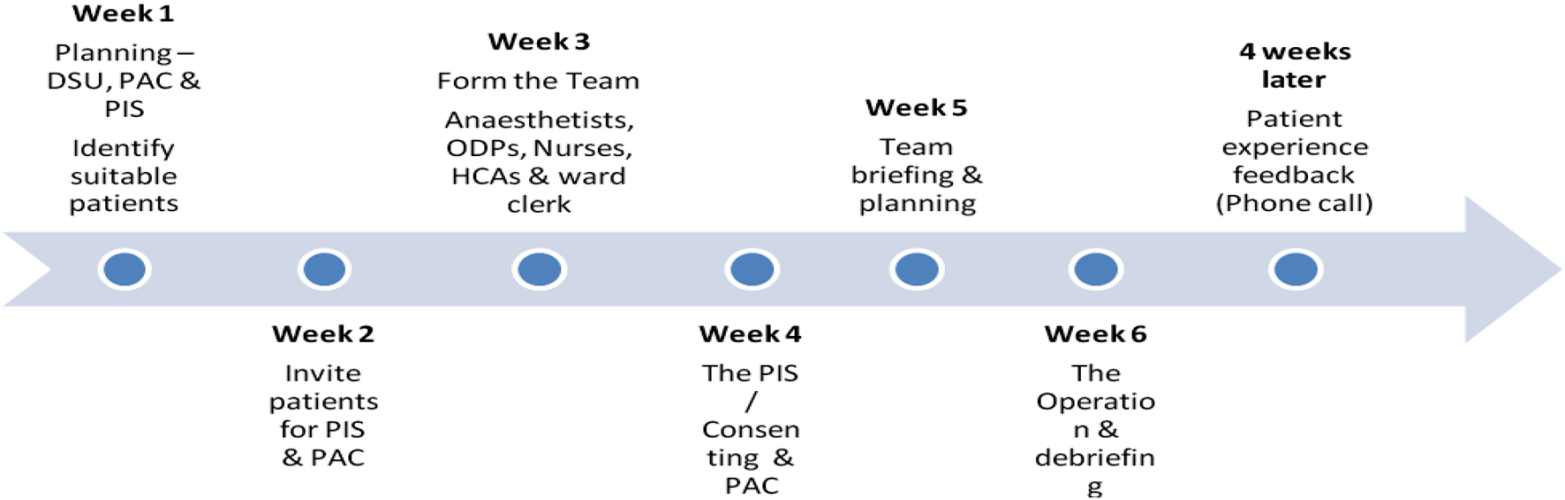


## Results

The improvements to the system, noted by the CAMP model, were recorded and documented using the SQUIRE guidelines. The impact of the CAMP was listed as per the following parameters-

1. *Waiting lists:* The two CAMPs between December 18 and March 19, operated upon 23 patients over 7 sessions (1 session = 4 h), viz 3 patients per session. This brought about a 40% increase in the overall productivity. This in regular repeated cycles of 3 months would hugely impact the waiting list to reduce the waiting times.

**Table:** 

Hernia CAMPs	Theatre setting	No. of theatre sessions	No. of patients operated	Improvement in no. of cases	Grade of surgeon
CAMP 1 (Dec 18)	Day surgery unit (DSU)	4	12	4	Fellows/SAS
CAMP 2 (Apr 19)	Main operating theatre (MOT)	3.5	11	4	Sr. registrar
2. *Theatre work-flow:* With a common theme, all the similar-sided hernias were put into one theatre. This helped minimise errors and maximise efficiency, with a 40% improvement in the overall turnover of the list.
3. *Patient involvement:* Appropriate patient selection is crucial to the success of the model. Only patients suitable for day procedures were invited to the information and consenting session. The pre-operative assessment was also done at the same time and it helped to build patient confidence and rapport. The peer support noticed through the CAMP was fantastic, as they were already familiar with their surgeons and their fellow patients. The post-op follow-up phone-call 4 weeks later documented an enhanced patient experience, with more than 95% satisfaction levels with the patients and approval of the improved pathway. It also reinforced our commitment and accountability to their care.
4. *Team building:* Identifying like-minded surgeons (non-consultant grades) and anaesthetists, with the support of the nurses, ODPs, HCAs, with admin staff, was vital. The opportunity to engage them early through the planning and having their input into the execution of the CAMP gave the ‘team’ a sense of ownership and boosted their morale with a greater purpose to work for. This was the highlight of the feedback from the participants of the CAMP.
  *Cost-effectiveness:* The improved efficiency and reduced operating times between the patients, improved the turn over which translated into more effective use of resources. The DSU is the most cost-effective place to operate on inguinal hernias.

**Table:** 

Category	Site (% of procedures done here)	Cost (GBP-average)	Income (GBP-average)	Theatre time (minutes-mean)	Length of stay (days-mean)
Elective	MOT (30%)	3222	1299	65	1
	DSU (50%)	2465	1399	65	0
Emergency	MOT (20%)	4004	2230	88	2
5. *Training:* The single-themed operating day with the right choice of patients also promises to be a great opportunity to train surgeons in training, as was demonstrated by Hernia CAMP-2, whereby a ST-7 registrar was supervised as the lead surgeon, by the senior fellow.
6. *Morbidity and mortality:* The singular theme and same-sided procedures, were an enhanced safety measure that minimised the risk of errors. The only variation to report was the over-night stay of two elderly men with urinary retention, requiring catheterisation (Clavien–Dindo grade 1) in the immediate post-op period. There was no other morbidity (nor mortality) reported at 4 weeks, as recorded by the telephonic follow-ups.

## Discussion

Every organisation differs in their systems and methods of workflow. Workflow patterns evolve by interactions within the system as conflicts and complexities arise. Likewise, there are patterns that can be designed based on priorities and local needs, which after integration evolve [2]. The CAMP model provides one such framework to improvise, improve and evolve. The impact is multi-fold on both the providers and the receivers of care.

Though surgeon-led, coordination between a multidisciplinary team, with nurses and support staff is vital to the success of these modelling processes as they hugely impact the execution, delivery and overall quality of care [3]. Irrespective of the size of the organisation and the process involved, clear communication is crucial. ‘Team-building’ is at the crux of these processes. The cycle of surgical process modelling and analysis paves the way for further optimisation and improvement of processes [4].

The hernia ‘CAMP’ demonstrated a model for improvement. The Hernia CAMP model was later adapted to laparoscopic Cholecystectomies with success. With similar adaptations to local needs, organisations can create their own solutions for their local problems. Lessons learnt from them will guide future improvements and execution. It needs a team of motivated people to lead and continually improvise based on the local circumstances.

The challenges in the success of the process are to get both consultants and the non-consultant surgeons to subscribe to it and cooperate through it. It can be a win–win on either side, depending on perspectives.

Camps are common in Asia and Africa, where they are conducted by a team with hundreds of patients. The inspired adaptation of this approach within the NHS needs its own modifications. Though the numbers can not be compared, the process could be made more efficient. Though set in a tertiary NHS teaching hospital, these lessons are transferable and adaptable into any organisation, despite its limitations.

The challenges posed to us in this resource-limited setting are universal. We need to be more creative in our approach to improvise. This will help us provide innovative solutions tailored to the local systems, to improve their efficiency.

## Conclusions

The Hernia ‘CAMP’ model offers an easily adaptable pathway to tackle the ever-growing waiting lists for open inguinal hernioplasties across the NHS trusts. It helps improve focus, morale and patient experience with a well-defined and concerted pathway. The DSU seems to be the most cost-effective and logistically viable environment.

With its adaptability to Cholecystectomies, it has shown the model to be transferable to various procedures, suitable for DSU settings with the appropriate expertise.

These quality improvement initiatives, offer an opportunity to non-consultant surgeons to step-up their responsibilities, while their consultant colleagues address other urgent and complex procedures. The CAMPs also provide an opportunity to train theme-based and build more confident trainees.

Regular need-based CAMPs with improvements made based on previous feedbacks, will lead to better processes of enhanced productivity, within an environment of collaboration and ownership. This is the need of the hour within the NHS.
